# Mealybug Population Dynamics: A Comparative Analysis of Sampling Methods for *Saccharicoccus sacchari* and *Heliococcus summervillei* in Sugarcane (*Saccharum* sp. Hybrids)

**DOI:** 10.3390/insects15070492

**Published:** 2024-07-01

**Authors:** Hang Xu, Jacob A. Humpal, Bree A. L. Wilson, Gavin J. Ash, Kevin S. Powell

**Affiliations:** 1 Centre for Crop Health, University of Southern Queensland, West Street, Toowoomba, QLD 4350, Australia; 2 Sugar Research Australia, 34 Hall Road, Gordonvale, QLD 4865, Australia; 3 Grains Research and Development Corporation, Herries Street, Toowoomba, QLD 4350, Australia; 4 Institute for Life Sciences and the Environment, University of Southern Queensland, West Street, Toowoomba, QLD 4350, Australia; 5 Sugar Research Australia, Queen Street, Brisbane, QLD 4000, Australia

**Keywords:** sugarcane pink mealybug, pasture mealybug, *Saccharum* spp., seasonal abundance, comparative trapping

## Abstract

**Simple Summary:**

Our research is the first comparative field-based study on (i) the above- and belowground population dynamics and seasonal abundance and (ii) the effectiveness of conventional and innovative sampling methods for two mealybug species, *Saccharicoccus sacchari* (Cockerell, 1895) (Hemiptera: Coccomorpha, Pseudococcidae) and *Heliococcus summervillei* (Brookes, 1978) (Hemiptera: Coccomorpha, Pseudococcidae), in commercial sugarcane (*Saccharum* sp. hybrids) (f. Poaceae) over consecutive growing seasons. The novel description of *S. sacchari* and *H. summervillei* on sugarcane in multiple seasons, along with the best sampling techniques, allows for accurate monitoring and comparison within the Pseudococcidae family, thus aiding the ability to monitor the pest status in commercial sugarcane.

**Abstract:**

This research is focused on a comparative field-based study of the population dynamics and sampling methods of two mealybug species, *Saccharicoccus sacchari* (Cockerell, 1895) (Hemiptera: Coccomorpha, Pseudococcidae) and *Heliococcus summervillei* (Brookes, 1978) (Hemiptera: Coccomorpha, Pseudococcidae), in sugarcane (*Saccharum* sp. hybrids) (f. Poaceae) over consecutive growing seasons. The research monitored and compared the above- and belowground populations and seasonal abundance of these two mealybug species in sugarcane fields in Far North Queensland, with non-destructive sampling techniques of yellow sticky traps, pan traps, and stem traps, and destructive sampling of the whole leaf and whole plant. The results indicated that *S. sacchari* (n = 29,137) was more abundant and detected throughout the growing season, with population peaks in the mid-season, while *H. summervillei* (n = 2706) showed peaks of the early-season activity. *S. sacchari* is primarily located on sugarcane stems and roots, compared to *H. summervillei*, which is located on leaves and roots. The whole-leaf collection and stem trap were the most effective sampling techniques for quantification of *H. summervillei* and *S. sacchari*, respectively. This study enhanced the understanding of *S. sacchari* and the first-ever record of *H. summervillei* on sugarcane in Australia and will contribute to the development of more effective pest management strategies.

## 1. Introduction

Sugarcane (*Saccharum* sp. hybrids) (f. Poaceae) is a vital cash crop globally, serving as a primary source for sugar and bioenergy production [1,2]. However, sustainable sugarcane cultivation faces challenges from various insect pests worldwide [3,4,5], including mealybugs (Hemiptera: Pseudococcidae). Thirty species of mealybug are known sugarcane pests globally [6], and some species also have the potential to vector viruses [7]. In Australia, sugarcane is attacked by the sugarcane pink mealybug, *Saccharicoccus sacchari* (Cockerell, 1895) (Hemiptera: Coccomorpha, Pseudococcidae), which is widely distributed in the majority of cane-growing countries [8,9] and overseas vectors of the Sugarcane bacilliform MO virus [10], which is not present in Australia. Although *S. sacchari* is considered a relatively minor pest in Australia [11], it has been associated with reduced sugar quality and secondary pathogen infection [12,13]. Despite the widespread distribution of *S. sacchari* in Australia, limited field-based research has been conducted on this species [8]. In contrast, the pasture mealybug, *Heliococcus summervillei* (Brookes, 1978) (Hemiptera: Coccomorpha, Pseudococcidae), has only recently been observed in sugarcane in Australia in 2021 (KS Powell, pers. comm); its pest status is undetermined, and field-based research is even more limited. Recent studies by Xu, et al. [14] suggest that *H. summervillei* may be associated with Yellow Canopy Syndrome (YCS), a condition first observed in Far North Queensland in 2012 that only affects sugarcane. Other studies by Hauxwell, et al. [15] suggest that it may be the causative pest for pasture dieback in grasses. Both *S. sacchari* and *H. summervillei* predominantly feed on the Poaceae family, including sugarcane. Overall, the comparison of the results of these two mealybug species is hampered by the paucity of published literature, especially on sugarcane.

### 1.1. Saccharicoccus sacchari

Sugarcane pink mealybug, *S. sacchari*, is a polyphagous pest that is found in 100 countries globally [16], including several sugarcane-growing countries. *Saccharicoccus sacchari* has piercing–sucking mouthparts and feeds on sugarcane plants, mainly on the stem under overlapping leaf sheaths and the root system, where it obtains essential nutrients from the phloem [17,18]. Significant worldwide damage has been reported by Atiqui and Murad [19] and Gamal El-Dein, Sanaa and Fatma [12]: high levels of *S. sacchari* affect both physical and chemical properties of the crop, including stalk weight decrease of up to 34% and sucrose (C_12_H_22_O_11_) reduction of 28% [20]. In Australia, the effect that large infestations of *S. sacchari* have on crop performance has seldom been quantified [8]. *Saccharicoccus sacchari* population levels are usually kept below the economic threshold [21], rarely necessitating control measures. However, indirect damage from *S. sacchari* includes the transmission of sugarcane bacilliform MO virus [10], previously known as sugarcane bacilliform virus [22], which has been reported in Australia, as well as in major sugarcane growing regions worldwide, including South Africa, India, China, the United States, and Cuba [23]. Virus infection can lead to significant losses in biomass production in susceptible sugarcane cultivars, including decreased juice sucrose content, gravity purity, and reduced stalk weight [24,25]. In addition, the presence of *S. sacchari* is associated with an increase in the incidence of red rot in sugarcane, a fungal disease caused by *Colletotrichum falcatum* Went (Glomerellales) [13], making *S. sacchari* an insect pest of concern.

*Saccharicoccus sacchari* is present year-round and colonizes both the roots and aerial stem tissue of sugarcane [8,26]. The generation time of *S. sacchari* ranges from 25 days at 30 °C to 107 days at 20 °C air temperature [27]. Their abundance increases as stem tissue develops [21], with *S. sacchari* generally reaching a maximum population in the early growing season (February to March in Australia) [28,29]. After harvest, *S. sacchari* survives on roots and emerges from the soil to re-establish aerial colonies once storage tissue is formed above ground level [9,21]. Bonnett and Hewitt [8] found that ratoon crops harbored more *S. sacchari* than that observed in plant cane. However, no previous research has effectively quantified the root-feeding underground population of *S. sacchari*.

Published methods for assessing *S. sacchari* populations include visual counting of *S. sacchari* on sugarcane stems. This is the standard but albeit labor-intensive sampling method to determine the aboveground population abundance [8,9,12,26]. The only study demonstrating the technique of assessing the underground *S. sacchari* population involved collecting roots with adhering soil, and then soaking and washing until all soil was removed and counting mealybugs [26].

### 1.2. Heliococcus summervillei

Summerville [30] first reported *H. summervillei* as a “mealy bug” on *Paspalum* grass in the Atherton Tablelands, Queensland, Australia. It has subsequently been found on grasses in other regions of Australia [31,32], India [32,33,34,35], New Caledonia [36], and Pakistan [31,32,35]. More recently, the first report of *H. summervillei* was observed in the Western Hemisphere [37], in Puerto Rico and Barbados. *Heliococcus summervillei* is a polyphagous pest that predominantly feeds on the Poaceae family [16], and its research in Australia has primarily focused on its suggested association with Pasture dieback [15,38,39,40], a condition that kills sown and native summer-growing pastures [41]. It has been reported to affect up to 4.4 million hectares in Queensland [42]. Pasture dieback was confirmed on the North Coast of New South Wales, Australia, in autumn 2020 [41]. In contrast, the biology, ecology, and sampling methods of *H. summervillei* and its association with sugarcane are largely undescribed, except for a recent study that indicates that *H. summervillei* may be associated with Yellow Canopy Syndrome (YCS) in sugarcane [14].

This is the first research that compares the population dynamics, seasonal abundance, and spatial distribution of *S. sacchari* and *H. summervillei* in sugarcane under field conditions over consecutive growing seasons. The study used both conventional and novel, and destructive and non-destructive sampling methods to determine the most effective and least labor-intensive system for monitoring both *S. sacchari* and *H. summervillei*. The development of species-specific monitoring approaches will ultimately lead to improved management strategies for each species.

## 2. Materials and Methods

### 2.1. Study Sites

In this research, all investigations took place on a commercial sugarcane farm in Far North Queensland, Australia. The study site was located between Gordonvale and Aloomba (17°06′09.7″ S 145°47′53.1″ E, 15 m.a.s.l). The seasonal activity of *H. summervillei* and *S. sacchari* was assessed over two successive sugarcane seasons on a single sugarcane variety, Q240. In season one, sampling was conducted between November 2021 and May 2022, from plant cane. In season two, sampling was from October 2022 to May 2023, on the first ratoon.

### 2.2. Experimental Design

**Figure 1:**
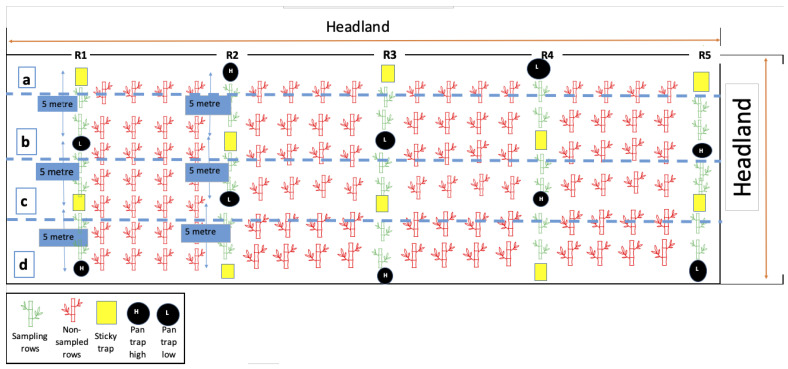
Schematic field trial design showing trap type and trap locations for monitoring mealybugs across two seasons in a commercial sugarcane block. Where R1 to R5 (green) are the sampling rows, and non-sampled adjacent rows (red). Four sampling points (**a**–**d**) are five meters apart in each sampling row, with sticky traps (yellow square) and pan traps (black cycle).

Five insect sampling methods were used to (i) characterize and quantify mealybug population abundance and seasonal activity and (ii) compare the performance and effectiveness of collecting mealybugs using selected sampling techniques, including yellow sticky traps, pan traps, stem traps, whole-leaf, and whole-plant collections. The trial layout design and sample locations for both seasons are shown schematically in Figure 1.

Within the sugarcane block, five sampling rows were selected (designated as R1 to R5), with a gap of four unsampled rows between each sampling row. Within each sampling row, four reference sampling points (a–d) were defined, each five meters apart, and they remained in the exact location throughout the sampling period. The position of the two yellow sticky-trap sample points (n = 10) and the two pan-trap sample points (n = 10) was alternately located in each of the five sample rows (Figure 1). Two stem traps were located at each sample reference point (n = 20). The yellow sticky traps, pan traps, and stem traps were installed at two heights, approximately 20 cm (designated as L = low) and 150 cm (designated as H = high).

### 2.3. Non-Destructive Sampling Methods

#### 2.3.1. Yellow Sticky Trap

**Figure 2:**
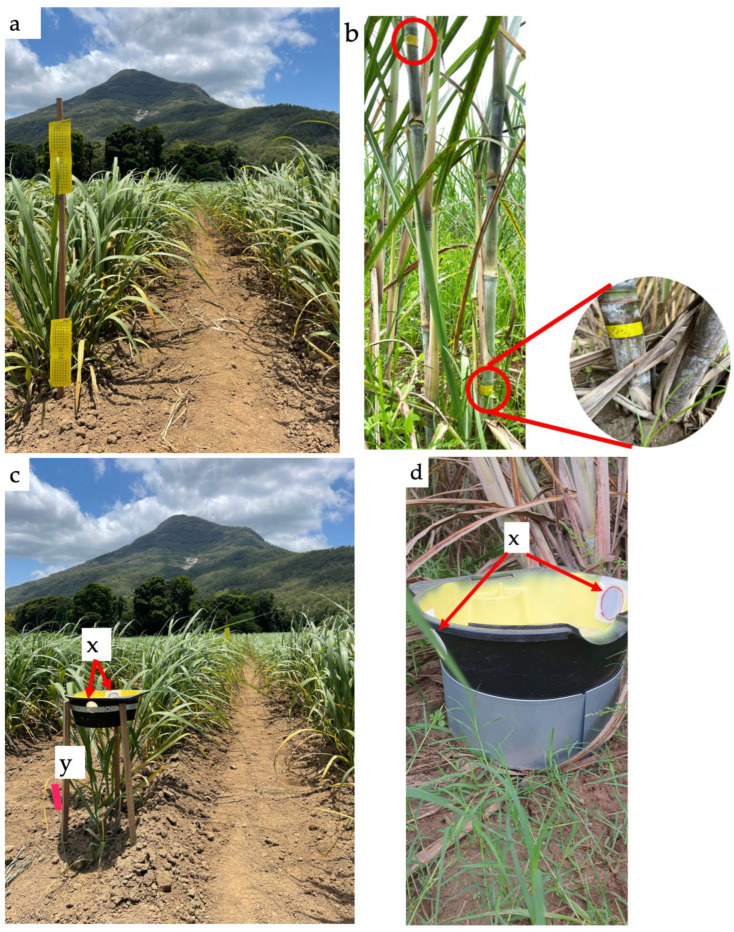

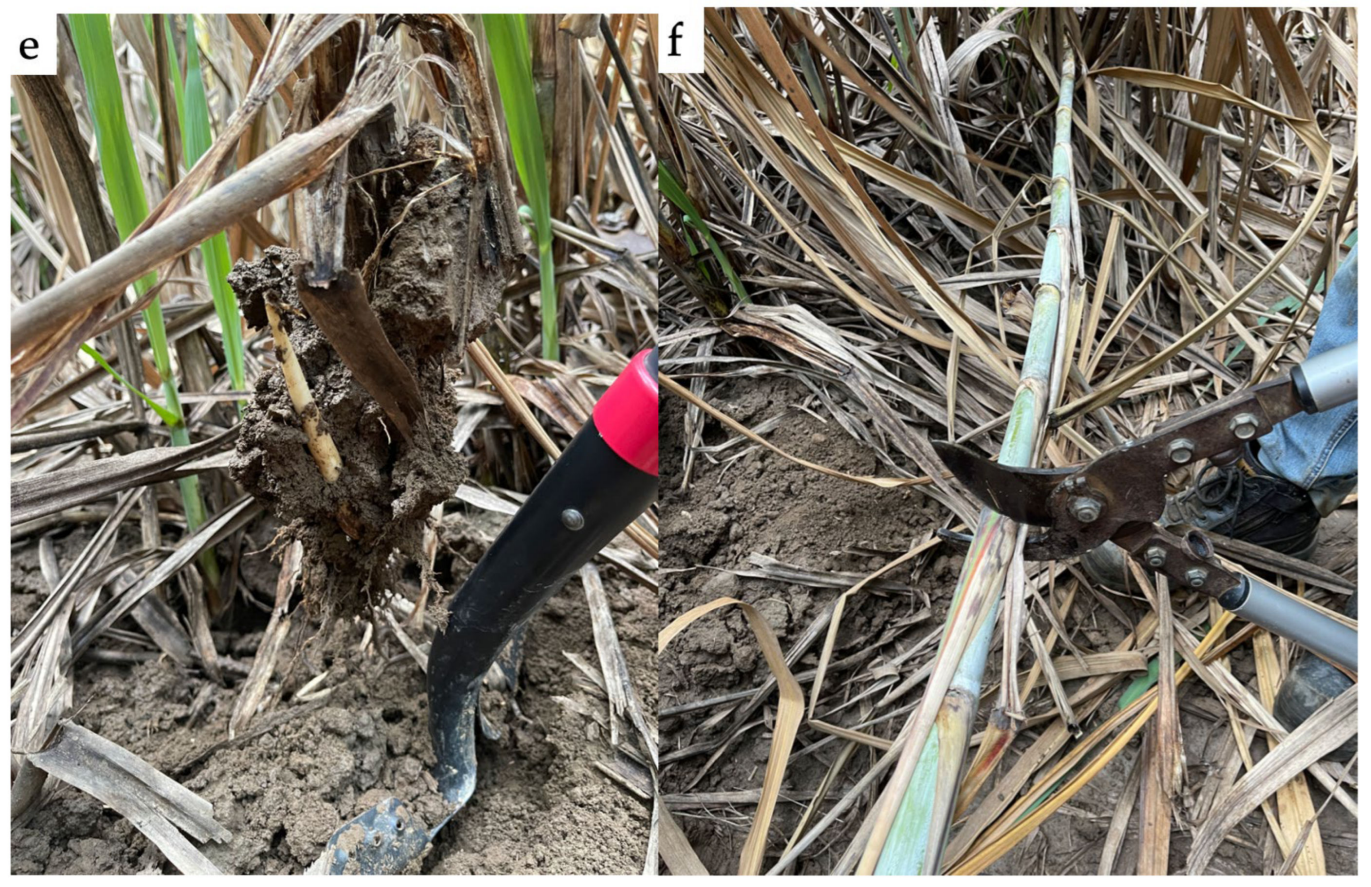
Mealybug sampling techniques used in sugarcane: yellow sticky trap (**a**); stem trap (high and low) (**b**); pan traps—high (**c**) and low (**d**); and whole-plant collection—digging (**e**) and cut into three sections (**f**). (x) Drainage holes (2 cm in diameter) sealed with fine mesh. (y) Stakes to stabilize pan trap (high). (z) Base of the pan trap (low) to stabilize and restrict movement of the traps.

The yellow sticky trap method was modified from Trębicki, et al. [43], who used it to monitor both wind-dispersed and alate (winged) invertebrates, whose active stages are predominately on the foliage. Commercially available yellow sticky traps (32 cm × 10 cm; Bugs for Bugs, Toowoomba, Australia) were attached to wooden stakes (2.5 × 2.5 × 150 cm; Growies, Ashmore, Australia) using a staple gun (Figure 2a). The traps were labelled with sample, row, and position numbers and set at two heights, sticky-L (low) at 20 cm above the soil level and sticky-H (high) at 150 cm above the soil level. At biweekly intervals, the sticky traps were removed and replaced with new sticky traps. Following this, the used sticky traps were placed in clear plastic bags (18 × 17 cm; Snap Lock^®^ Reseal Bags—Sandwich, GLAD, Sydney, Australia) to prevent cross-contamination during transport and storage. Samples were stored at 25 °C and examined for the presence of mealybugs using a low-power binocular microscope (Leica^®^) at 64×, 160×, and 400× magnification.

#### 2.3.2. Stem Trap

Stem traps were modified based on Powell, et al. [44] and were used to monitor crawling invertebrates whose active stages are predominately on the plant stem. A stem trap consisted of 18 mm wide yellow electrical tape coated with Tanglefoot^®^ (The Tanglefoot Company, Grand Rapids, MI, USA) wrapped around two adjacent sugarcane stems, with two height variants at each sample point (Figure 2b). Stem-H (high) was positioned at 150 cm, and Stem-L (low) was placed 20 cm above the soil level to account for differences in the upward vertical dispersal of wingless crawling invertebrates on the sugarcane stem. The traps were collected fortnightly and replaced with fresh stem traps. The used traps were placed into clear plastic bags (35.5 × 40.5 cm × 40 μm; Magic Seal Bags, Venus Packaging, Australia), stored at 25 °C, and examined for the presence of invertebrates using a low-power binocular microscope, as described previously.

#### 2.3.3. Pan Trap

Pan or Moericke traps [45] are designed to monitor alate and wind-dispersed invertebrates. This study used modified pan traps to quantify mealybugs that are predominately near either the base or the middle of the sugarcane canopy. Pan traps were constructed using a black plastic bowl (10 cm depth × 40 cm diameter; Oil Drain Pan, Super Cheap Auto, Brisbane, Australia) spray-painted yellow (Gloss Dandelion Yellow; Dura Max, Dulux, Melbourne, Australia). All pan traps were filled to near capacity with water, and a few drops of dishwater detergent (Dishwashing Liquid, Glitz, Sydney, Australia) were added as a surfactant to prevent insect escape. Two height variants of pan trap were installed: Pan-H (high) (Figure 2c) was placed 150 cm above the soil level, sitting on three wooden stakes and a steel-strip constructed stand; and Pan-L (low) (Figure 2d) was placed 20 cm above soil level, sitting on an interlocking steel tree ring (30 cm diameter; Whites, Australia). An additional modification was made to the pan traps by introducing two mesh-covered outlet holes (4 cm diameter) to release excess water without losing invertebrates during high rainfall events. The contents of each trap were collected biweekly by straining the contents through a fine sieve (22 cm diameter; New Challenger, Myanmar). Mealybugs were transferred from the sieve to a sealable plastic container and stored at 1–5 °C before separation and identification of invertebrates microscopically.

### 2.4. Destructive Sampling Methods

Whole-leaf and whole-plant collection are destructive methods for counting plant-dwelling invertebrates that involve removing specimens from collected plant material, before storage, and placing them into tubes (2 mL; screw cap micro tube, Sarstedt, Adelaide, Australia).

#### 2.4.1. Whole Leaves

Whole-leaf collection was used to monitor relatively small sessile invertebrates whose active stages are predominately on the leaf surface and are generally apterous adult females and nymphs. Biweekly, ten leaves were collected randomly in the five sampling rows. Samples were stored in labelled 2 L round screw-top plastic containers (13 cm diameter × 20.3 cm height; Target, Australia) and then stored at 1–5 °C before processing.

#### 2.4.2. Whole-Plants

Whole-plant collection was used to quantify and characterize mealybugs whose active stages show zonal preference in multiple zones from the root to the meristem. A single sugarcane plant was randomly selected and removed fortnightly from each of the five sample rows, digging around the root zone (30–50 cm deep) to remove the whole plant with root and attached soil (Figure 2e). The sugarcane plant was then cut into three zonal sections (Figure 2f): section one was the belowground root zone; section two was the low-to-middle canopy, including stem and foliage (the first 50% of the sugarcane plant aboveground); and section three was the higher canopy, including stem, foliage, and meristem (the second 50% of the sugarcane plant aboveground). Mealybug removal from the root section was performed by modifying the flotation method [26] into (i) soaking the root with tap water for 30 min in a plastic container (23.8 × 23.8 × 22.8 cm; Super Storer™ Bulk Food Container 8.5 L, Décor, Dandenong South, Australia); (ii) decanting the water through two layers of sieves—top = a 1680 μm aperture sieve, and bottom = a 250 μm aperture sieve; (iii) washing the root in the container until all adhering soil was removed, and then the excess water was rinsed through the sieves; and (iv) the remaining insect materials in the top and bottom sieves were contained in Petri dishes for microscope examination. Samples in plant sections two and three were stored in labelled resealable plastic bags (35.5 × 40.5 cm × 40 μm; Magic Seal Bags, Venus Packaging, Richmond, Australia) in a refrigerator (1–5 °C) before being processed for qualitative and quantitative analysis of mealybugs microscopically. All plant samples were oven-dried at 70 °C for 24 h after examination to calculate the number of mealybugs per g dry weight of plant matter.

### 2.5. Mealybug Identification

The mealybug specimen was slide-mounted [46] and identified using microscopy and identification keys [47,48,49]. Additional preparation via the removal of Tanglefoot^®^ [50] was also carried out, where the specimen was collected from sticky traps and stem traps.

### 2.6. Statistical Analysis

Statistical analysis was conducted using SPSS Statistics (Version 29.0.2.0; IBM Corp, Armonk, NY, USA). A Kruskal–Wallis test was used to compare differences between the numbers of *S. sacchari* and *H. summervillei* trapped using non-destructive and destructive sampling techniques in both seasons.

## 3. Results

### 3.1. Overall Seasonal Activity

Overall, mealybug abundance during season 1 (November 2021 to May 2022) was almost fourfold lower than in season 2 (October 2022 to May 2023) (Table 1). The number of *S. sacchari* (n = 29,137) caught for both seasons was tenfold higher than the number of *H. summervillei* (n = 2706), indicating that *S. sacchari* was the predominant mealybug species present.

**Figure 3:**
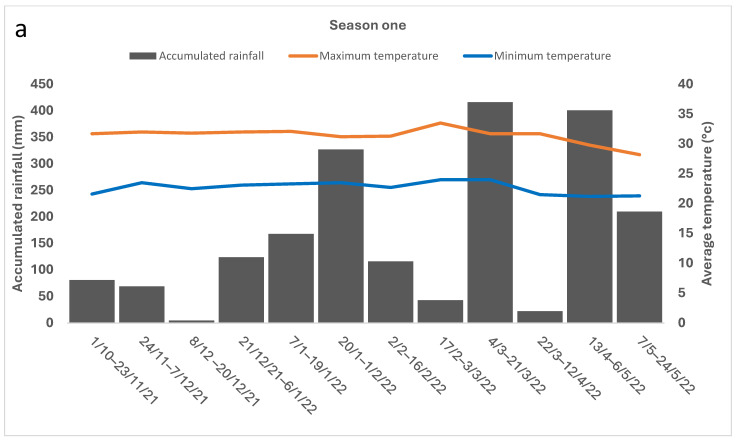

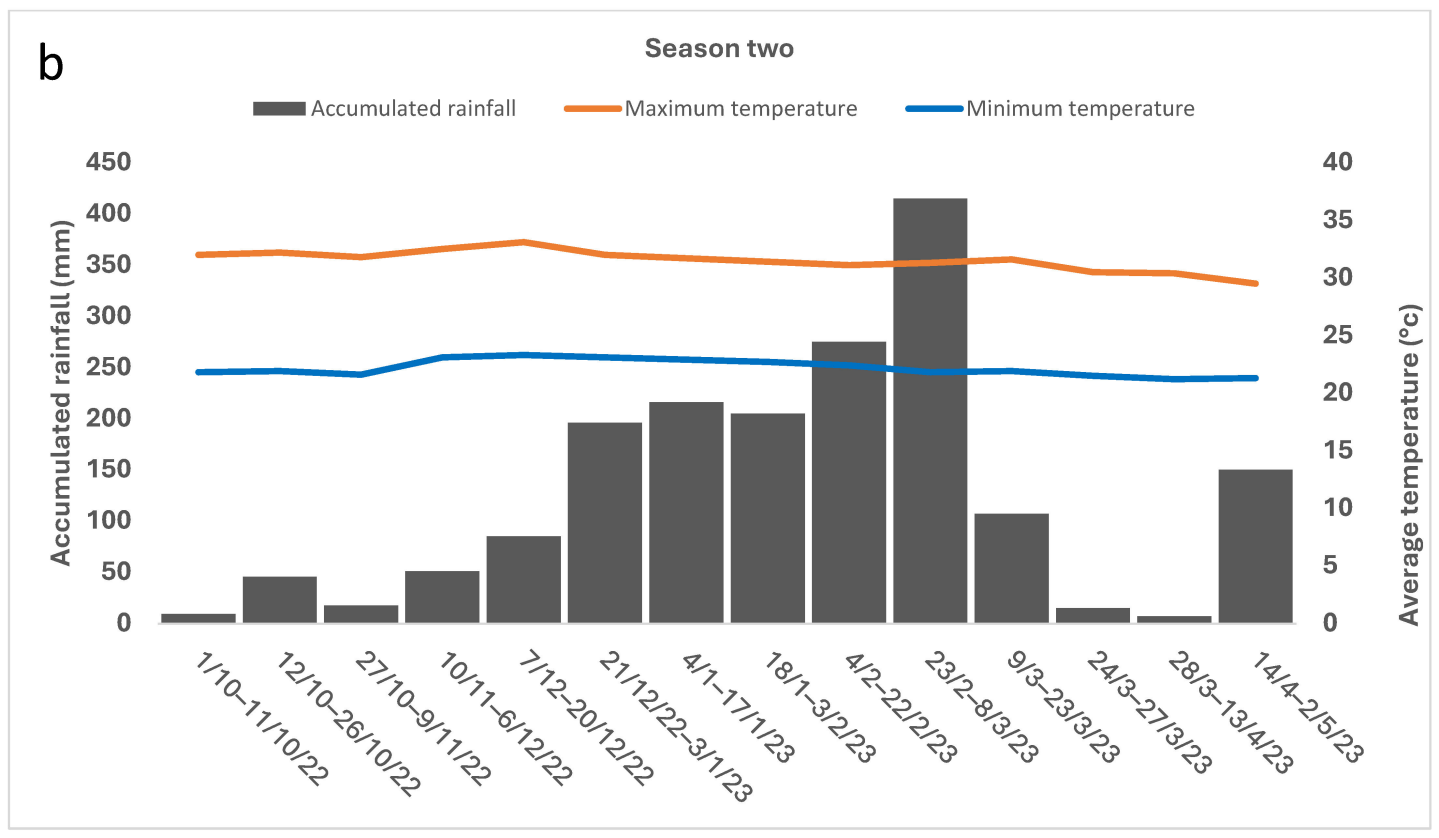
Accumulated rainfall distribution and average maximum and minimum air temperature of (**a**) season one and (**b**) season two over two consecutive sampling seasons: October 2021 to May 2022 and October 2022 to May 2023.

Rainfall was only marginally higher (<10%) in season one than in season two. Comparing season two to season one, we noted that *H. summervillei* populations appeared to decline substantially (a 3-fold decrease). In contrast, the *S. sacchari* population increased fourfold in season two. The rainfall-accumulation data (Figure 3) showed general trends across both sampling seasons: In the early season (October to early January), the rainfall was approximately 200 mm or less. The mid-season (late January to April) had more than 400 mm of rainfall events, and the late-season (May to June) rainfall data were between 100 and 200 mm. The average air temperature showed minimal fluctuation for both seasons (Figure 3). The average maximum temperature was 30 °C ± 3 °C, and the average minimum temperature was 22 °C ± 3 °C.

### 3.2. Trap Evaluation

**Figure 4:**
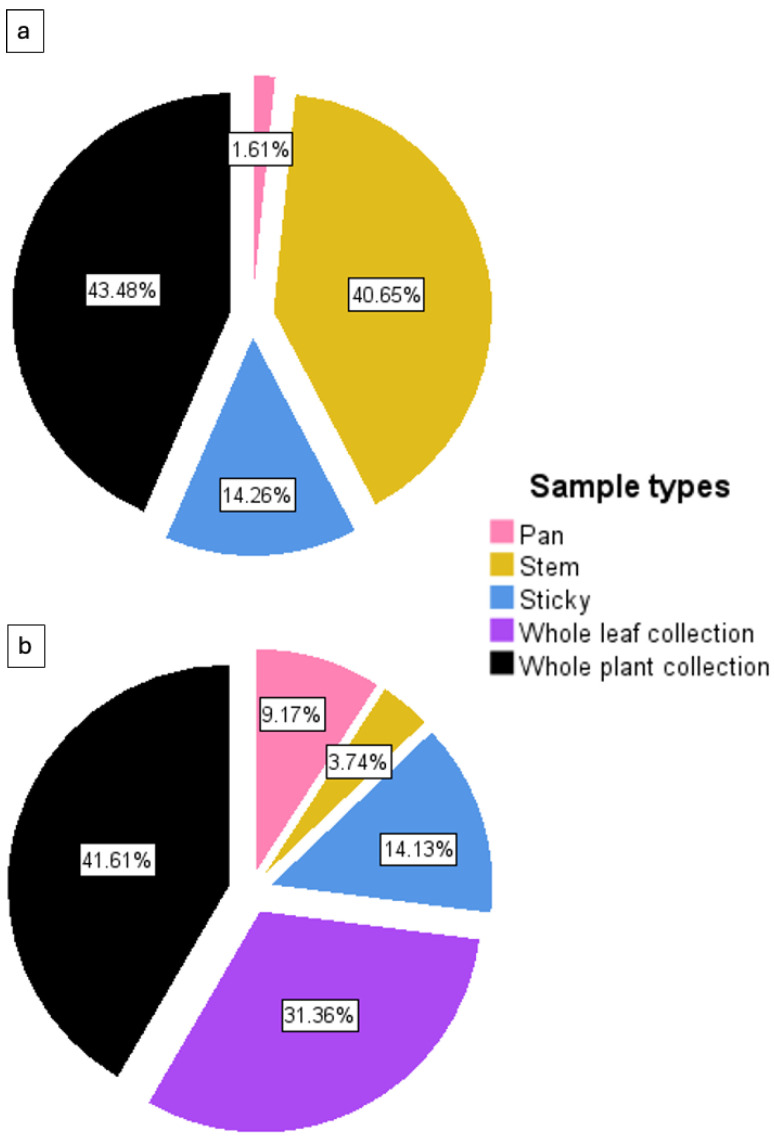
Total abundance of (**a**) *S. sacchari* and (**b**) *H. summervillei* over two seasons, using three non-destructive (pan, stem, and sticky) and two destructive (whole-leaf collection and whole-plant collection) sampling methods. Showing pooled data.

After two consecutive seasons of in-field collection and 27 sampling dates, a comparative performance analysis of five different sampling methods—pan traps, stem traps, sticky traps, whole-leaf collections, and whole-plant collections—was conducted (Figure 4 and Table 2). Since the destructive whole-leaf and whole-plant collections were one-off sampling events, their data were analyzed separately rather than cumulatively, using non-destructive traps. The trap data for the two mealybug species were separated into two categories for statistical comparison: destructive trapping methods (whole-leaf and whole-plant collections) and non-destructive trapping methods (pan traps, stem traps, and yellow sticky traps).

It was apparent that the sampling method affected the mealybug species collected. In regard to destructive sampling methods, the whole-plant method was the most effective method for collecting both mealybug species. The whole-leaf was effective for collecting *H. summervillei* (Figure 4b), but no *S. sacchari* was collected using the whole-leaf collection.

Non-destructive stem traps and destructive whole-plant collections trapped more than 80% of the *S. sacchari.* In contrast, sticky traps collected a similar proportion of *H. summervillei* (14.1%) and *S. sacchari* (14.2%). Pan traps were the least effective sample method for both species, collecting only 9.2% and 1.6% of *H. summervillei* and *S. sacchari*, respectively.

### 3.3. Population Dynamics

**Figure 5:**
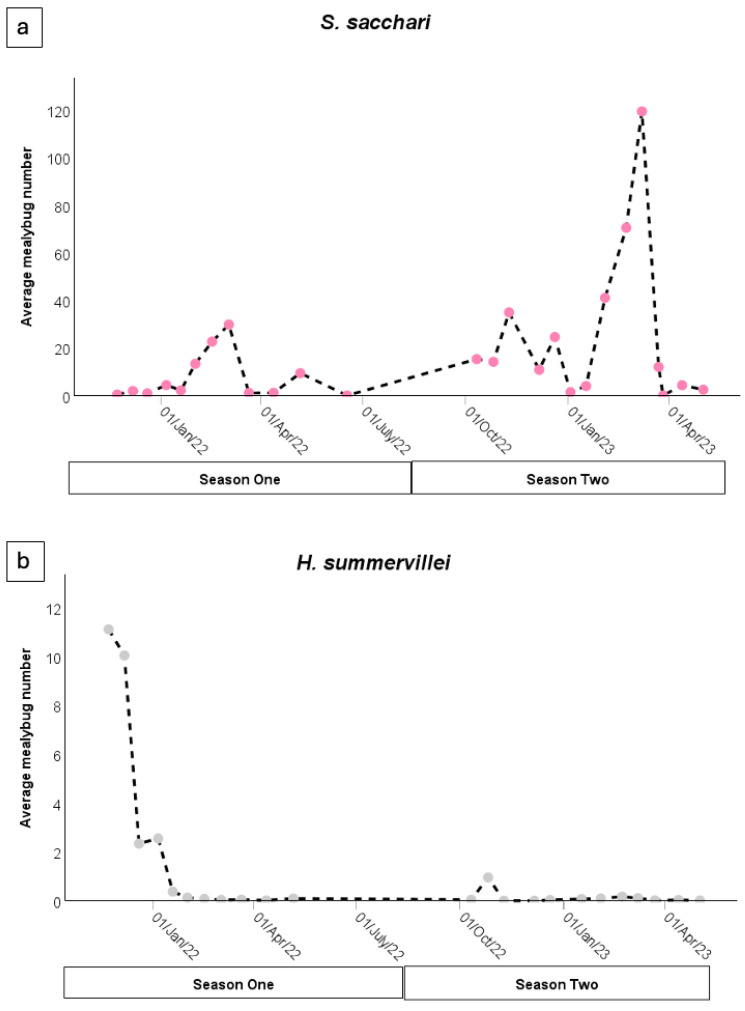
The average number of (**a**) *S. sacchari* in stem traps and whole-plant collections and (**b**) *H. summervillei* in whole-leaf and whole-plant collections sampled on each sampling date over two consecutive seasons (season one: October 2021 to May 2022 and season two: October 2022 to May 2023). Showing pooled data.

Irrespective of trap type, *S. sacchari* showed a bimodal population peak in season one (2021–2022) and a trimodal population peak in season two (2022–2023) (Figure 5a). However, only the *S. sacchari* sampled in February had a significant difference (*p* ≤ 0.017) compared to other sampling months in both seasons. In season two (2022–2023), earlier in the sampling period (October to December), there was a ninefold-higher abundance of *S. sacchari* compared to season one (2021–2022).

*Heliococcus summervillei* had a unimodal peak (Figure 5b) in both the first and the second seasons. A significantly (*p* < 0.01) higher abundance of *H. summervillei* was trapped earlier in the first season (November to December 2021) compared to the rest of the sampling period (January to May 2022). In both seasons, population peaks occurred in the early season (pre-January).

### 3.4. Saccharicoccus sacchari

**Figure 6:**
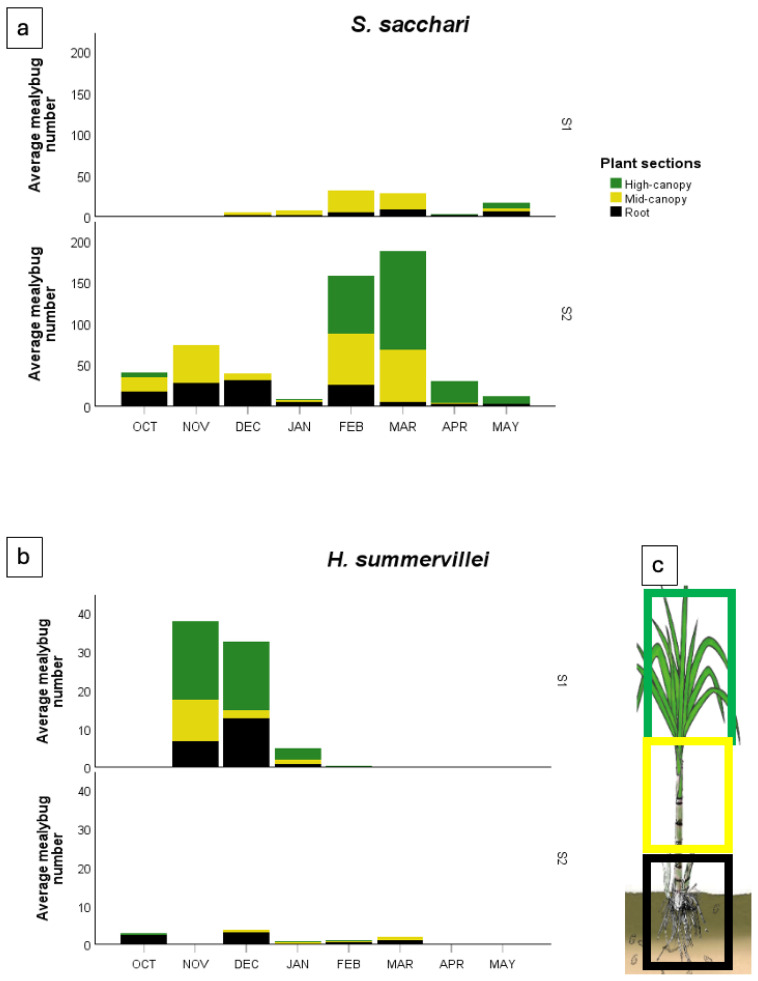
Zonal distribution of (**a**) *S. sacchari* and (**b**) *H. summervillei* in season one (S1), 2021–2022 (**top**); and season two (S2), 2022–2023 (**bottom**), on sampled sections of the whole-plant collection. (**c**) Schematic zonal sections in the whole-plant collection showing three key sections: black, the root; yellow, first 50% of plant aboveground; green, second 50% of plant aboveground. Showing pooled data.

For *S. sacchari*, the population abundance, as assessed using destructive methods, showed seasonal differences in zonal abundance (Figure 6a). In season one, the optimal sampling periods were from January to March, and the populations were predominantly collected in the mid-canopy section on the stems. Although populations were relatively low in the root zone, they were evident in the root system from November to May.

In season two, overall population abundance was higher than in season one. This species could be detected in all three zonal sections sampled. In the root zone section, *S. sacchari* were detected from October to May, with the highest abundance recorded from October to February. In the ground-level-to-mid-canopy region, there were two abundance peaks in the early season: October to December; and mid-season, from February to March. In the mid-to-top canopy region, abundance was the highest from February to April, suggesting migration of the mealybugs gradually from the base to the top canopy region during the season.

The root section had more *S. sacchari* than other sugarcane sections at the beginning of the season (December, season one; and October, season two) and at the end of the season (May in season one). The greatest abundance of *S. sacchari* occurred in the mid-canopy section in February and March for both seasons. The number of *S. sacchari* in the high-canopy region was greater from February to April in season two.

### 3.5. Heliococcus summervillei

For *H. summervillei*, the population abundance assessed using destructive sampling methods showed seasonal differences in zonal abundance (Figure 6b). In season one, the optimal sampling periods were from November to January. In early season one (November and December), more activity occurred in the high-canopy region of sugarcane plants, and the *H. summervillei* population decreased after January 2022. The population of *H. summervillei* was relatively low across season two and was predominantly on the root system from October to March.

## 4. Discussion

The objective of this research was to determine the optimal sampling methods for *S. sacchari* and *H. summervillei*. This was performed by also comparing the *S. sacchari* and *H. summervillei* above- and belowground population dynamics and quantifying seasonal abundance under field conditions.

Although the population dynamics of mealybugs on sugarcane have been examined previously in Australia and overseas, most of these limited studies have focused on a single mealybug species, *S. sacchari*, and predominantly its aboveground population, as assessed using a single destructive sampling method [8,9,12,29]. In contrast, our research is the first comparative field-based study to be conducted in commercially grown sugarcane on two mealybug species, *H. summervillei* and *S. sacchari*, across successive seasons, using both destructive and non-destructive sampling methods. We compared the above- and belowground population dynamics and seasonal abundance of these insects. We also tested the effectiveness of sampling methods specifically for *H. summervillei* and *S. sacchari* over two seasons, using a total of five methods in two categories: non-destructive—yellow sticky traps, pan traps, and stem traps; and destructive—whole-leaf and whole-plant collection. In addition, vacuum sampling and sweep net were also trialed during the two seasons. However, the data are not presented here due to the relatively low catch numbers overall and their relative ineffectiveness in capturing mealybugs.

*Saccharicoccus sacchari* and *H. summervillei* were recorded in both seasons. *Heliococcus summervillei* showed peak activity in the early season and was predominantly active on sugarcane leaves. By contrast, *S. sacchari* was active underground on sugarcane roots in the early season, gradually migrating to the aboveground stem tissue, with peak abundance occurring in the mid-season. Furthermore, whole-leaf collection for *H. summervillei* and the stem trap method for *S. sacchari* were the preferred sampling methods in terms of effectiveness and reduced labor and time inputs.

The total abundance of *S. sacchari* and *H. summervillei* differed markedly over the two seasons. *S. sacchari* was much more abundant in sugarcane than *H. summervillei*. Even though the seasonal rainfall was less than a 10% difference between the two seasons, *S. sacchari* had a higher abundance in ratoon crops (season 2022–2023) than the plant canes (season 2021–2022). This is similar to seasonal trends observed by Bonnett and Hewitt [8]. In stark contrast, *H. summervillei* had a relatively high abundance in the 2021–2022 season, followed by reduced activity in the 2022–2023 season. Also (refer to Figure 3), the air temperature during the sugarcane growing period was suitable for *S. sacchari* in a short generation time [27]. We speculate that *S. sacchari* is a mealybug species well evolved to sugarcane and out-competes *H. summervillei* to survive on sugarcane. Above the ground, *S. sacchari* feeds on stem tissue beneath the leaf sheaths, giving it more protection than *H. summervillei*, which feeds on the back of the leaf blade, except during extreme weather events, such as tropical cyclones. Below ground, *S. sacchari* has a higher abundance than *H. summervillei* on the roots after harvesting and is capable of rapid population growth on sugarcane in tropical field conditions [27]. When comparing Figure 6a,b, we speculate that *H. summervillei* out-competes *S. sacchari* in regard to space and other resources possibly because *S. sacchari* has a higher female fecundity. However, further studies are required to confirm this.

Sampling methods have been developed previously for studies on the population dynamics of *S. sacchari* in sugarcane fields [8,26,51]. However, their use by the sugarcane industry has been limited, as they are mainly destructive and both labor- and time-intensive. In our study for *S. sacchari*, one destructive method, whole-plant sampling, and one non-destructive method, stem trap sampling, were identified as the most suitable to characterize relative abundance during the growing season. Stem trap sampling has not been used previously to study *S. sacchari*, and this represents an improvement on previous sampling approaches for this species. Although highly effective in sampling for *S. sacchari*, these two methods were relatively ineffective in sampling *H. summervillei*. The non-destructive stem sampling method has only previously been used to successfully monitor the dispersal of root-feeding Hemiptera, namely first instar grape phylloxera *Daktulosphaira vitifoliae* in vineyards [52]. The research presented here offers a novel and user-friendly system compared to the conventional destructive method that growers or extension staff currently use to monitor *S. sacchari*. As neither of the *S. sacchari* and *H. summervillei* collected has an alate form, it is unsurprising that yellow sticky traps and pan traps were relatively ineffective sampling methods, and it was evident that the mealybug life stages collected were wind-dispersed nymphs. The higher abundance of *H. summervilleri* in pan traps is most likely because they are present on leaves, and they become more easily dislodged from leaves by rain and wind dispersal than the stem-dwelling *S. sacchari*, which were not observed on leaves.

Like previous studies, we observed that the peak abundance of *S. sacchari* occurred from February to March, during the growing season. However, in contrast to previous studies, this is the first study to quantify the belowground population dynamics of *S. sacchari* by collecting sugarcane roots in two consecutive seasons. We also identified the seasonal migration/spatial migration of *S. sacchari* that occurred on sugarcane. In the early season, the abundance of *S. sacchari* occurred on the sugarcane roots. During the season, the abundance of *S. sacchari* gradually shifts to stems: from the lower canopy to mid-canopy and even higher cane canopy. The total number of *S. sacchari* increased significantly and reached its peak abundance in the mid-season. At the end of the season, *S. sacchari* aggregated back to the sugarcane roots. At the same time, its aboveground population decreased sharply, and this decrease may have been caused by natural enemies [53].

In the case of *H. summervillei*, the most effective sampling methods were whole-plant and whole-leaf collection. The least destructive of these two methods is the whole-leaf collection, and, consequently, a more straightforward visual assessment method of *H. summervillei* on leaves could also be developed. Sugarcane recovers effectively from leaf removal due to its rapid canopy growth during the season. In contrast, *S. sacchari* was not observed on any leaf samples; this is likely because *S. sacchari* is known to be an exclusive stem and root feeder, whereas *H. summervillei* is known to be an exclusive leaf and root feeder. Prior to this study, no sampling methods were described for *H. summervillei* on sugarcane; and methods have only recently been developed for pasture grasses [15].

In summary, this paper contributed to the existing body of knowledge on *S. sacchari* and *H. summervillei* by comparing the population dynamics and developing user-friendly sampling methods for sugarcane. Through a nuanced examination, we aspire to provide valuable insights for researchers, policymakers, and practitioners engaged in sugarcane pest management.

## Figures and Tables

**Table 1:** Rainfall and total abundance of two mealybug species, *Saccharicoccus sacchari* and *Heliococcus summervillei*, trapped over two consecutive sugarcane-crop seasons.

Season	*S. sacchari*	*H. summervillei*	Rainfall (mm)
2021–2022	5654	2089	1984
2022–2023	23,483	617	1833
Total	29,137	2706	3817

Showing pooled data from all sampling methods.

**Table 2:** Pairwise comparisons (Kruskal–Wallis Test) of mealybug abundance data using non-destructive and destructive sampling methods over two sugarcane seasons: 2021–2022 (season one) and 2022–2023 (season two).

Season 1 and Season 2		*S. sacchari*	*H. summervillei*
Non-destructive	Pan–Stem	<0.001 ***	<0.001 ***
	Pan–Sticky	0.328	<0.001 ***
	Stem–Sticky	<0.001 ***	<0.001 ***
Destructive	Whole leaf–Whole plant	<0.001 ***	0

*** *p* < 0.001.

## Data Availability

The data presented in this study are available on request from the corresponding author due to on-going publication.
